# (4*R*)-Ethyl 4-(4-chloro­phen­yl)-2-hydr­oxy-5-oxo-2,3,4,5-tetra­hydro­pyrano[3,2-*c*]chromene-2-carboxyl­ate

**DOI:** 10.1107/S1600536809051976

**Published:** 2009-12-19

**Authors:** Yifeng Wang, Wei Zhang, Xiangsheng Xu, Guangcun Zhang

**Affiliations:** a State Key Laboratory Breeding Base of Green Chemistry-Synthesis Technology, Zhejiang University of Technology, Hangzhou, 310014, People’s Republic of China

## Abstract

The title compound, C_21_H_17_ClO_6_, is optically pure and adopts an *R* configuration. It was obtained by an organocatalytic asymmetric Michael addition of 4-hydroxy­coumarin with (*E*)-ethyl 4-(4-chloro­phen­yl)-2-oxobut-3-enoate. The structure consists of a tetra­hydro­pyran unit fused to the coumarin ring  ring system. The hydroxyl and phenyl groups are on the same side of the tetra­hydro­pyrane ring. The benzene ring is almost perpendicular to the coumarin ring [dihedral angle of 72.89 (3)°]. In the crystal structure, inter­molecular O—H⋯O hydrogen bonds are observed. An intra­molecular O—H⋯O contact also occurs.

## Related literature

For general background to the use of coumarin derivatives as intermediates in organic and natural product synthesis, see: Fylaktakidou *et al.*, (2004 ▶); Hoult *et al.*, (1996 ▶). For a related structure, see: Zhang *et al.* (2009 ▶).
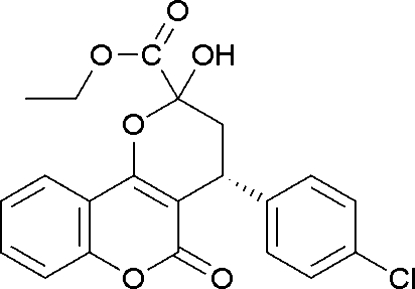


## Experimental

### 

#### Crystal data

C_21_H_17_ClO_6_
*M* *_r_* = 400.80
Monoclinic, 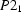

*a* = 5.4818 (3) Å
*b* = 14.8358 (7) Å
*c* = 11.3403 (6) Å
β = 94.6807 (15)°
*V* = 919.20 (8) Å^3^
*Z* = 2
Mo *K*α radiation
μ = 0.25 mm^−1^
*T* = 296 K
0.37 × 0.31 × 0.08 mm

#### Data collection

Rigaku RAXIS-RAPID diffractometer
Absorption correction: multi-scan (*ABSCOR*; Higashi, 1995 ▶) *T*_min_ = 0.905, *T*_max_ = 0.981
8978 measured reflections
3606 independent reflections
3027 reflections with *I* > 2σ(*I*)
*R*_int_ = 0.025

#### Refinement

*R*[*F* ^2^ > 2σ(*F* ^2^)] = 0.031
*wR*(*F* ^2^) = 0.080
*S* = 1.00
3606 reflections
256 parameters
1 restraint
H-atom parameters constrained
Δρ_max_ = 0.16 e Å^−3^
Δρ_min_ = −0.20 e Å^−3^
Absolute structure: Flack (1983 ▶), 1434 Friedel pairs
Flack parameter: 0.07 (6)

### 

Data collection: *PROCESS-AUTO* (Rigaku, 2006 ▶); cell refinement: *PROCESS-AUTO*; data reduction: *CrystalStructure* (Rigaku, 2007 ▶); program(s) used to solve structure: *SHELXS97* (Sheldrick, 2008 ▶); program(s) used to refine structure: *SHELXL97* (Sheldrick, 2008 ▶); molecular graphics: *ORTEP-3 for Windows* (Farrugia,1997 ▶); software used to prepare material for publication: *WinGX* (Farrugia, 1999 ▶).

## Supplementary Material

Crystal structure: contains datablocks global, I. DOI: 10.1107/S1600536809051976/zq2019sup1.cif

Structure factors: contains datablocks I. DOI: 10.1107/S1600536809051976/zq2019Isup2.hkl

Additional supplementary materials:  crystallographic information; 3D view; checkCIF report

## Figures and Tables

**Table 1:** Hydrogen-bond geometry (Å, °)

*D*—H⋯*A*	*D*—H	H⋯*A*	*D*⋯*A*	*D*—H⋯*A*
O4—H4⋯O2^i^	0.82	2.27	2.9184 (19)	136
O4—H4⋯O5	0.82	2.19	2.671 (2)	118

Symmetry code: (i) 
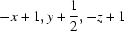
.

**Fig. 1.**
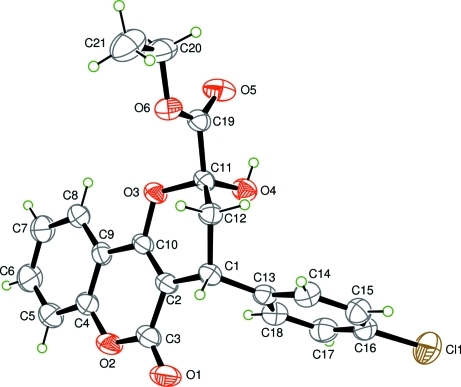
The asymmetric unit of the structure of the title compound, with the atomic labeling scheme. Displacement ellipsoids are drawn at the 50% probability level.

**Fig. 2.**
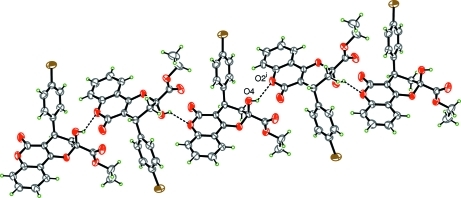
The molecular packing of the title compound showing H-bridge interactions.

## supplementary crystallographic information

### Comment

Coumarin derivatives are common found in a variety of natural products, and are used as versatile intermediates in organic and natural product synthesis (Fylaktakidou *et al.*, 2004; Hoult *et al.*, 1996). The title compound could be synthesized through an asymmetric Michael addition of 4-hydroxycoumarin with (*E*)-ethyl 4-(4-chlorophenyl)-2-oxobut-3-enoate, catalyzed by a tertiary-amine-squaramide catalyst. As part of our study in organocatalysis, the absolute structure of the title compound was determined, which adopts a *R* configuration. The structure consists of a tetrahydropyrane fused beside the coumarin ring. The hydroxyl and phenyl groups are on the same side of the tetrahydropyrane ring. The benzene ring is almost perpendicular to the coumarin ring with a dihedral angle of 72.89 (3)° between the mean planes. In addition, intermolecular O—H···O hydrogen bonds are observed in the crystal structure.

### Experimental

A mixture of 4-hydroxycoumarin (0.1 mmol), (*E*)-ethyl 4-(4-chlorophenyl)-2-oxobut-3-enoate 2 (0.1 mmol) and the catalyst 3-((1*S*) -(6-methoxyquinolin-4-yl)(8-vinylquinuclidin-2-yl)methylamino) -4-((*R*)-1-phenylethylamino)cyclobut-3-ene-1,2-dione (0.0025 mmol) in ClCH_2_CH_2_Cl (1.0 ml) was stirred at room temperature for 3 h (monitored by TLC). The mixture was purified by column chromatography on silica gel, eluted by petroleum ether/EtOAc (10:1 to 3:1) to give the desired Michael adducts. Suitable crystals of the title compound were obtained by slow evaporation of a mixture solution of CH_2_Cl_2_ and *i*PrOH at room temperature.

### Refinement

All hydrogen atoms were refined in calculated positions with C—H = 0.98 Å(*sp*), C—H = 0.97 Å (*sp^2^*), C—H = 0.96 Å (*sp^3^*), C—H = 0.93 Å (aromatic), O—H = 0.82 Å, and with *U*_iso_(H) = 1.2*U*_eq_ of the carrier atoms.

### Crystal data

**Table:**

C_21_H_17_ClO_6_	*F*(000) = 416
*M**_r_* = 400.80	*D*_x_ = 1.448 Mg m^−^^3^
Monoclinic, *P*2_1_	Mo *K*α radiation, λ = 0.71073 Å
Hall symbol: P 2yb	Cell parameters from 7553 reflections
*a* = 5.4818 (3) Å	θ = 3.3–27.4°
*b* = 14.8358 (7) Å	µ = 0.25 mm^−^^1^
*c* = 11.3403 (6) Å	*T* = 296 K
β = 94.6807 (15)°	Platelet, colorless
*V* = 919.20 (8) Å^3^	0.37 × 0.31 × 0.08 mm
*Z* = 2	

### Data collection

**Table:**

Rigaku RAXIS-RAPID diffractometer	3606 independent reflections
Radiation source: rolling anode	3027 reflections with *I* > 2σ(*I*)
graphite	*R*_int_ = 0.025
Detector resolution: 10.00 pixels mm^-1^	θ_max_ = 27.4°, θ_min_ = 3.3°
ω scans	*h* = −7→6
Absorption correction: multi-scan (*ABSCOR*; Higashi, 1995)	*k* = −19→16
*T*_min_ = 0.905, *T*_max_ = 0.981	*l* = −14→14
8978 measured reflections	

### Refinement

**Table:**

Refinement on *F*^2^	Hydrogen site location: inferred from neighbouring sites
Least-squares matrix: full	H-atom parameters constrained
*R*[*F*^2^ > 2σ(*F*^2^)] = 0.031	*w* = 1/[σ^2^(*F*_o_^2^) + (0.0403*P*)^2^ + 0.110*P*] where *P* = (*F*_o_^2^ + 2*F*_c_^2^)/3
*wR*(*F*^2^) = 0.080	(Δ/σ)_max_ < 0.001
*S* = 1.00	Δρ_max_ = 0.16 e Å^−^^3^
3606 reflections	Δρ_min_ = −0.20 e Å^−^^3^
256 parameters	Extinction correction: *SHELXL*, Fc^*^=kFc[1+0.001xFc^2^λ^3^/sin(2θ)]^-1/4^
1 restraint	Extinction coefficient: 0.014 (2)
Primary atom site location: structure-invariant direct methods	Absolute structure: Flack (1983), 1434 Friedel pairs
Secondary atom site location: difference Fourier map	Flack parameter: 0.07 (6)

### Special details

**Table:**

Geometry. All e.s.d.'s (except the e.s.d. in the dihedral angle between two l.s. planes) are estimated using the full covariance matrix. The cell e.s.d.'s are taken into account individually in the estimation of e.s.d.'s in distances, angles and torsion angles; correlations between e.s.d.'s in cell parameters are only used when they are defined by crystal symmetry. An approximate (isotropic) treatment of cell e.s.d.'s is used for estimating e.s.d.'s involving l.s. planes.
Refinement. Refinement of *F*^2^ against ALL reflections. The weighted *R*-factor *wR* and goodness of fit *S* are based on *F*^2^, conventional *R*-factors *R* are based on *F*, with *F* set to zero for negative *F*^2^. The threshold expression of *F*^2^ > σ(*F*^2^) is used only for calculating *R*-factors(gt) *etc*. and is not relevant to the choice of reflections for refinement. *R*-factors based on *F*^2^ are statistically about twice as large as those based on *F*, and *R*- factors based on ALL data will be even larger.

### Fractional atomic coordinates and isotropic or equivalent isotropic displacement parameters (Å^2^)

**Table:**

	*x*	*y*	*z*	*U*_iso_*/*U*_eq_	
C19	0.7695 (3)	0.87640 (13)	0.68614 (17)	0.0368 (4)	
O6	0.9549 (3)	0.87424 (10)	0.76723 (12)	0.0456 (3)	
O5	0.6200 (3)	0.93532 (10)	0.67137 (15)	0.0542 (4)	
C20	0.9886 (5)	0.95257 (18)	0.8444 (2)	0.0577 (6)	
H20A	0.8533	0.9579	0.8940	0.069*	
H20B	0.9965	1.0072	0.7978	0.069*	
C21	1.2228 (5)	0.9388 (2)	0.9187 (2)	0.0677 (7)	
H21A	1.3564	0.9380	0.8690	0.081*	
H21B	1.2169	0.8825	0.9600	0.081*	
H21C	1.2458	0.9871	0.9749	0.081*	
Cl1	0.78443 (13)	0.72613 (6)	−0.01510 (5)	0.0714 (2)	
O3	0.6428 (2)	0.72598 (9)	0.68482 (11)	0.0369 (3)	
C9	0.5060 (3)	0.57839 (13)	0.71703 (16)	0.0345 (4)	
O4	0.6019 (2)	0.80479 (9)	0.50872 (12)	0.0396 (3)	
H4	0.5259	0.8519	0.5155	0.047*	
C10	0.6604 (3)	0.63786 (12)	0.65397 (16)	0.0325 (4)	
O2	0.6645 (3)	0.45423 (9)	0.60916 (14)	0.0467 (4)	
O1	0.9452 (3)	0.47428 (10)	0.48350 (16)	0.0577 (5)	
C14	1.0913 (3)	0.72144 (15)	0.31531 (17)	0.0407 (4)	
H14	1.2357	0.7420	0.3552	0.049*	
C13	0.9207 (3)	0.67714 (13)	0.37856 (16)	0.0334 (4)	
C2	0.8067 (3)	0.60639 (12)	0.57288 (16)	0.0335 (4)	
C1	0.9793 (3)	0.66573 (12)	0.51166 (16)	0.0332 (4)	
H1	1.1406	0.6370	0.5222	0.040*	
C8	0.3482 (4)	0.60857 (15)	0.79984 (18)	0.0429 (5)	
H8	0.3403	0.6695	0.8184	0.051*	
C15	1.0524 (4)	0.73588 (16)	0.19483 (18)	0.0459 (5)	
H15	1.1688	0.7657	0.1540	0.055*	
C5	0.3671 (5)	0.42524 (15)	0.7439 (2)	0.0547 (6)	
H5	0.3721	0.3644	0.7248	0.066*	
C11	0.7569 (3)	0.79097 (12)	0.60903 (16)	0.0320 (4)	
C6	0.2158 (5)	0.45589 (17)	0.8250 (2)	0.0603 (7)	
H6	0.1184	0.4149	0.8618	0.072*	
C16	0.8385 (4)	0.70536 (14)	0.13638 (18)	0.0438 (5)	
C17	0.6664 (4)	0.65979 (16)	0.19587 (19)	0.0476 (5)	
H17	0.5232	0.6388	0.1554	0.057*	
C3	0.8158 (4)	0.51022 (13)	0.54999 (19)	0.0421 (5)	
C12	1.0021 (3)	0.75658 (12)	0.57806 (17)	0.0334 (4)	
H12A	1.1086	0.7491	0.6500	0.040*	
H12B	1.0763	0.8007	0.5289	0.040*	
C4	0.5140 (4)	0.48726 (14)	0.69059 (19)	0.0414 (5)	
C18	0.7094 (3)	0.64567 (14)	0.31706 (18)	0.0409 (4)	
H18	0.5944	0.6146	0.3573	0.049*	
C7	0.2036 (4)	0.54659 (17)	0.8539 (2)	0.0544 (6)	
H7	0.0988	0.5658	0.9093	0.065*	

### Atomic displacement parameters (Å^2^)

**Table:**

	*U*^11^	*U*^22^	*U*^33^	*U*^12^	*U*^13^	*U*^23^
C19	0.0424 (10)	0.0289 (9)	0.0403 (10)	−0.0053 (9)	0.0112 (8)	0.0000 (8)
O6	0.0582 (8)	0.0365 (8)	0.0417 (8)	−0.0048 (7)	0.0013 (6)	−0.0088 (6)
O5	0.0549 (9)	0.0375 (8)	0.0713 (11)	0.0070 (7)	0.0109 (8)	−0.0129 (7)
C20	0.0693 (14)	0.0498 (14)	0.0545 (14)	−0.0138 (12)	0.0078 (11)	−0.0206 (11)
C21	0.0677 (15)	0.081 (2)	0.0540 (15)	−0.0195 (15)	0.0045 (12)	−0.0199 (14)
Cl1	0.0961 (5)	0.0781 (5)	0.0382 (3)	0.0081 (4)	−0.0048 (3)	0.0010 (3)
O3	0.0452 (7)	0.0252 (6)	0.0418 (7)	−0.0039 (6)	0.0128 (5)	0.0007 (6)
C9	0.0382 (9)	0.0321 (10)	0.0327 (9)	−0.0038 (8)	0.0006 (7)	0.0047 (8)
O4	0.0399 (7)	0.0346 (8)	0.0433 (7)	0.0059 (6)	−0.0023 (6)	−0.0007 (6)
C10	0.0375 (9)	0.0238 (8)	0.0355 (9)	−0.0013 (7)	−0.0008 (7)	0.0009 (7)
O2	0.0616 (9)	0.0260 (7)	0.0541 (9)	−0.0058 (6)	0.0140 (7)	−0.0031 (6)
O1	0.0745 (11)	0.0333 (8)	0.0688 (11)	0.0064 (8)	0.0263 (9)	−0.0074 (7)
C14	0.0350 (9)	0.0441 (11)	0.0432 (10)	−0.0005 (9)	0.0046 (8)	−0.0034 (10)
C13	0.0341 (8)	0.0285 (9)	0.0378 (10)	0.0050 (8)	0.0046 (7)	−0.0021 (8)
C2	0.0368 (9)	0.0256 (8)	0.0377 (10)	0.0003 (8)	0.0015 (8)	0.0005 (8)
C1	0.0315 (8)	0.0297 (9)	0.0383 (10)	0.0030 (8)	0.0024 (7)	0.0011 (8)
C8	0.0523 (12)	0.0357 (10)	0.0410 (11)	−0.0040 (9)	0.0060 (9)	0.0030 (8)
C15	0.0470 (11)	0.0466 (12)	0.0450 (11)	0.0027 (10)	0.0091 (9)	0.0025 (10)
C5	0.0785 (15)	0.0323 (11)	0.0547 (14)	−0.0159 (11)	0.0145 (12)	0.0029 (10)
C11	0.0353 (8)	0.0246 (9)	0.0361 (9)	−0.0023 (8)	0.0028 (7)	0.0018 (7)
C6	0.0808 (17)	0.0471 (14)	0.0556 (14)	−0.0241 (12)	0.0221 (12)	0.0057 (11)
C16	0.0553 (12)	0.0407 (12)	0.0353 (10)	0.0113 (9)	0.0035 (9)	−0.0019 (8)
C17	0.0456 (11)	0.0477 (13)	0.0479 (12)	0.0046 (11)	−0.0057 (9)	−0.0086 (10)
C3	0.0492 (12)	0.0293 (10)	0.0481 (12)	−0.0005 (9)	0.0062 (9)	0.0004 (9)
C12	0.0320 (8)	0.0309 (9)	0.0374 (10)	−0.0034 (7)	0.0033 (7)	−0.0021 (7)
C4	0.0518 (11)	0.0322 (10)	0.0400 (11)	−0.0070 (9)	0.0027 (9)	0.0008 (8)
C18	0.0377 (10)	0.0389 (11)	0.0461 (11)	−0.0004 (9)	0.0043 (8)	−0.0044 (9)
C7	0.0644 (14)	0.0506 (13)	0.0506 (13)	−0.0086 (12)	0.0198 (11)	0.0060 (10)

### Geometric parameters (Å, °)

**Table:**

C19—O5	1.201 (3)	C14—H14	0.9300
C19—O6	1.314 (2)	C13—C18	1.384 (3)
C19—C11	1.538 (3)	C13—C1	1.527 (3)
O6—C20	1.457 (3)	C2—C3	1.452 (3)
C20—C21	1.491 (3)	C2—C1	1.503 (3)
C20—H20A	0.9700	C1—C12	1.544 (2)
C20—H20B	0.9700	C1—H1	0.9800
C21—H21A	0.9600	C8—C7	1.389 (3)
C21—H21B	0.9600	C8—H8	0.9300
C21—H21C	0.9600	C15—C16	1.376 (3)
Cl1—C16	1.747 (2)	C15—H15	0.9300
O3—C10	1.359 (2)	C5—C6	1.366 (4)
O3—C11	1.466 (2)	C5—C4	1.393 (3)
C9—C4	1.386 (3)	C5—H5	0.9300
C9—C8	1.401 (3)	C11—C12	1.506 (2)
C9—C10	1.451 (3)	C6—C7	1.388 (4)
O4—C11	1.379 (2)	C6—H6	0.9300
O4—H4	0.8200	C16—C17	1.381 (3)
C10—C2	1.352 (3)	C17—C18	1.391 (3)
O2—C4	1.378 (2)	C17—H17	0.9300
O2—C3	1.385 (3)	C12—H12A	0.9700
O1—C3	1.202 (3)	C12—H12B	0.9700
C14—C15	1.382 (3)	C18—H18	0.9300
C14—C13	1.389 (3)	C7—H7	0.9300
			
O5—C19—O6	126.54 (19)	C7—C8—H8	120.3
O5—C19—C11	121.49 (18)	C9—C8—H8	120.3
O6—C19—C11	111.95 (17)	C16—C15—C14	118.80 (19)
C19—O6—C20	116.98 (18)	C16—C15—H15	120.6
O6—C20—C21	106.9 (2)	C14—C15—H15	120.6
O6—C20—H20A	110.3	C6—C5—C4	118.5 (2)
C21—C20—H20A	110.3	C6—C5—H5	120.8
O6—C20—H20B	110.3	C4—C5—H5	120.8
C21—C20—H20B	110.3	O4—C11—O3	108.53 (14)
H20A—C20—H20B	108.6	O4—C11—C12	111.09 (15)
C20—C21—H21A	109.5	O3—C11—C12	110.24 (14)
C20—C21—H21B	109.5	O4—C11—C19	110.03 (15)
H21A—C21—H21B	109.5	O3—C11—C19	102.12 (14)
C20—C21—H21C	109.5	C12—C11—C19	114.36 (15)
H21A—C21—H21C	109.5	C5—C6—C7	121.7 (2)
H21B—C21—H21C	109.5	C5—C6—H6	119.2
C10—O3—C11	116.05 (14)	C7—C6—H6	119.2
C4—C9—C8	119.26 (17)	C15—C16—C17	121.02 (19)
C4—C9—C10	117.16 (18)	C15—C16—Cl1	119.04 (17)
C8—C9—C10	123.56 (18)	C17—C16—Cl1	119.94 (16)
C11—O4—H4	109.5	C16—C17—C18	119.28 (18)
C2—C10—O3	124.48 (16)	C16—C17—H17	120.4
C2—C10—C9	121.85 (17)	C18—C17—H17	120.4
O3—C10—C9	113.68 (16)	O1—C3—O2	116.45 (18)
C4—O2—C3	121.76 (15)	O1—C3—C2	125.38 (19)
C15—C14—C13	121.85 (17)	O2—C3—C2	118.17 (17)
C15—C14—H14	119.1	C11—C12—C1	111.80 (14)
C13—C14—H14	119.1	C11—C12—H12A	109.3
C18—C13—C14	118.06 (17)	C1—C12—H12A	109.3
C18—C13—C1	124.12 (17)	C11—C12—H12B	109.3
C14—C13—C1	117.82 (15)	C1—C12—H12B	109.3
C10—C2—C3	119.47 (17)	H12A—C12—H12B	107.9
C10—C2—C1	122.87 (17)	O2—C4—C9	121.55 (17)
C3—C2—C1	117.45 (17)	O2—C4—C5	117.07 (19)
C2—C1—C13	115.59 (15)	C9—C4—C5	121.4 (2)
C2—C1—C12	108.35 (15)	C13—C18—C17	120.96 (19)
C13—C1—C12	112.80 (15)	C13—C18—H18	119.5
C2—C1—H1	106.5	C17—C18—H18	119.5
C13—C1—H1	106.5	C8—C7—C6	119.9 (2)
C12—C1—H1	106.5	C8—C7—H7	120.1
C7—C8—C9	119.3 (2)	C6—C7—H7	120.1
			
O5—C19—O6—C20	−2.5 (3)	O6—C19—C11—O3	79.70 (17)
C11—C19—O6—C20	178.81 (17)	O5—C19—C11—C12	141.84 (19)
C19—O6—C20—C21	−173.86 (19)	O6—C19—C11—C12	−39.4 (2)
C11—O3—C10—C2	11.5 (3)	C4—C5—C6—C7	0.7 (4)
C11—O3—C10—C9	−168.76 (14)	C14—C15—C16—C17	−1.0 (3)
C4—C9—C10—C2	−0.1 (3)	C14—C15—C16—Cl1	178.24 (17)
C8—C9—C10—C2	−178.63 (19)	C15—C16—C17—C18	0.8 (3)
C4—C9—C10—O3	−179.89 (16)	Cl1—C16—C17—C18	−178.46 (16)
C8—C9—C10—O3	1.6 (3)	C4—O2—C3—O1	177.8 (2)
C15—C14—C13—C18	1.2 (3)	C4—O2—C3—C2	−2.0 (3)
C15—C14—C13—C1	−179.16 (19)	C10—C2—C3—O1	−177.1 (2)
O3—C10—C2—C3	178.07 (17)	C1—C2—C3—O1	−2.2 (3)
C9—C10—C2—C3	−1.7 (3)	C10—C2—C3—O2	2.7 (3)
O3—C10—C2—C1	3.5 (3)	C1—C2—C3—O2	177.59 (16)
C9—C10—C2—C1	−176.26 (16)	O4—C11—C12—C1	−60.5 (2)
C10—C2—C1—C13	−114.25 (19)	O3—C11—C12—C1	59.81 (19)
C3—C2—C1—C13	71.1 (2)	C19—C11—C12—C1	174.18 (15)
C10—C2—C1—C12	13.4 (2)	C2—C1—C12—C11	−44.01 (19)
C3—C2—C1—C12	−161.21 (16)	C13—C1—C12—C11	85.26 (18)
C18—C13—C1—C2	7.8 (3)	C3—O2—C4—C9	0.2 (3)
C14—C13—C1—C2	−171.77 (17)	C3—O2—C4—C5	179.2 (2)
C18—C13—C1—C12	−117.64 (19)	C8—C9—C4—O2	179.47 (18)
C14—C13—C1—C12	62.8 (2)	C10—C9—C4—O2	0.9 (3)
C4—C9—C8—C7	0.1 (3)	C8—C9—C4—C5	0.5 (3)
C10—C9—C8—C7	178.6 (2)	C10—C9—C4—C5	−178.09 (19)
C13—C14—C15—C16	0.0 (3)	C6—C5—C4—O2	−180.0 (2)
C10—O3—C11—O4	79.38 (18)	C6—C5—C4—C9	−0.9 (3)
C10—O3—C11—C12	−42.5 (2)	C14—C13—C18—C17	−1.5 (3)
C10—O3—C11—C19	−164.42 (14)	C1—C13—C18—C17	178.97 (18)
O5—C19—C11—O4	16.0 (2)	C16—C17—C18—C13	0.5 (3)
O6—C19—C11—O4	−165.19 (15)	C9—C8—C7—C6	−0.3 (3)
O5—C19—C11—O3	−99.1 (2)	C5—C6—C7—C8	−0.1 (4)

### Hydrogen-bond geometry (Å, °)

**Table:**

*D*—H···*A*	*D*—H	H···*A*	*D*···*A*	*D*—H···*A*
O4—H4···O2^i^	0.82	2.27	2.9184 (19)	136
O4—H4···O5	0.82	2.19	2.671 (2)	118

Symmetry codes: (i) −*x*+1, *y*+1/2, −*z*+1.
