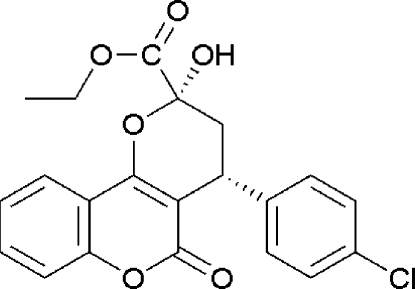# (4*R*)-Ethyl 4-(4-chloro­phen­yl)-2-hydr­oxy-5-oxo-2,3,4,5-tetra­hydro­pyrano[3,2-*c*]chromene-2-carboxyl­ate. Corrigendum

**DOI:** 10.1107/S1600536810002266

**Published:** 2010-01-30

**Authors:** Yifeng Wang, Wei Zhang, Xiangsheng Xu, Guangcun Zhang

**Affiliations:** a State Key Laboratory Breeding Base of Green Chemistry-Synthesis Technology, Zhejiang University of Technology, Hangzhou, 310014, People’s Republic of China

## Abstract

The absolute configuration in the title of the paper by Wang, Zhang, Xu & Zhang [*Acta Cryst.* (2010), E**66**, o217] is corrected.

## Abstract 2

Corrigendum to *Acta Cryst.* (2010), E**66**, o217.

In the paper by Wang *et al.* (2010) ▶, the chemical name given in the *Title* should be ‘(2*R*,4*R*)-Ethyl 4-(4-chloro­phen­yl)-2-hydr­oxy-5-oxo-2,3,4,5-tetra­hydro­pyrano[3,2-*c*]chro­mene-2-carboxyl­ate’. The absolute configuration was established by anomalous-dispersion effects in diffraction measurements on the crystal. The revised scheme is shown below.